# Risk factors, clinical features and outcome of new-onset acute kidney injury among critically ill patients: a database analysis based on prospective cohort study

**DOI:** 10.1186/s12882-021-02503-x

**Published:** 2021-08-25

**Authors:** Yi-Jia Jiang, Xiu-Ming Xi, Hui-Miao Jia, Xi Zheng, Mei-Ping Wang, Wen Li, Wen-Xiong Li

**Affiliations:** 1 Department of Surgical Intensive Critical Unit, Beijing Chao-yang Hospital, Capital Medical University, 8 Gongren Tiyuchang Nanlu, Chaoyang District, 100020 Beijing, China; 2 Department of Critical Care Medicine, Fuxing Hospital, Capital Medical University, Beijing, China; 3 Department of Epidemiology and Health Statistics, School of Public Health, Capital Medical University, Beijing, China

**Keywords:** Acute kidney injury, New-onset, Incidence, Risk factors, Outcome

## Abstract

**Background:**

Acute kidney injury (AKI) newly-emerged in intensive care unit (ICU), has not been thoroughly studied in previous researches, is likely to differ from AKI developed before ICU admission. This study aimed to evaluate the incidence, risk factors, clinical features and outcome of new-onset AKI in critically ill patients.

**Methods:**

The data of present study derived from a multicenter, prospective cohort study in17 Chinese ICUs (January 2014 - August 2015). The incidence, risk factors, clinical features and survival analysis of new-onset AKI were assessed.

**Results:**

A total of 3374 adult critically ill patients were eligible. The incidence of new-onset AKI was 30.0 % (*n* = 1012). Factors associated with a higher risk of new-onset AKI included coronary heart disease, hypertension, chronic liver disease, use of nephrotoxic drugs, sepsis, SOFA score, APACHEII score and use of vasopressors. The new-onset AKI was an independent risk factor for 28-day mortality (adjusted hazard ratio, 1.643; 95 % CI, 1.370–1.948; *P* < 0.001). 220 (21.7 %) patients received renal replacement therapy (RRT), 71 (32.3 %) of them were successfully weaning from RRT. More than half of the new-onset AKI were transient AKI (renal recovery within 48 h). There was no statistical relationship between transient AKI and 28-day mortality (hazard ratio, 1.406; 95 % CI, 0.840–1.304; *P* = 0.686), while persistent AKI (non-renal recovery within 48 h) was strongly associated with 28-day mortality (adjusted hazard ratio, 1.486; 95 % CI, 1.137–1.943; *P* < 0.001).

**Conclusions:**

New-onset AKI is common in ICU patients and is associated with significantly higher 28-day mortality. Only persistent AKI, but not transient AKI is associated with significantly higher 28-day mortality.

**Supplementary Information:**

The online version contains supplementary material available at 10.1186/s12882-021-02503-x.

## Background

Acute kidney injury (AKI) occurs in more than 50 % of intensive care patients and is associated with increased risks of in-hospital mortality and long-term chronic kidney disease [1–3]. AKI caused by diverse exposures and susceptible factors may present different incidence and prognosis [4]. Most AKI appears in the first 72 h after patients are transferred to intensive care unit (ICU) [5–7], but some critically ill patients may already have kidney damage before admission to ICU. Patients who developed AKI before ICU are likely to differ from those who develop AKI later on the following days. The appearance time of kidney injury implies the difference in causes and severity of the disease, which results in different clinical manifestations and outcomes. However, few studies have defined the exact time when AKI appears.

The severity of AKI is usually graded by the classifications such as the risk, injury, failure, loss of kidney function, and end-stage renal disease (RIFLE), acute kidney injury network (AKIN) and kidney disease improving global outcomes (KDIGO) staging system. More severe AKI is associated with poorer outcomes. However, these classification systems all evaluate the magnitude of serum creatinine increase and/or decrease in urine output, without considering factors such as underlying diseases and duration of AKI. Lately, the duration of kidney injury has been identified as an additional important dimension of AKI severity [8–13]. Transient AKI, typically defined as the duration of kidney injury less than 48–72 h, has been recognized with a lower risk of long-term adverse outcomes compare to AKI of longer duration [9–13], while its impact on short-term outcomes remains controversial in different clinical settings. Therefore, to evaluate the clinical characteristics and prognosis of new-onset AKI according to the duration of renal injury in ICU, we performed a retrospective analysis based on a database of a large, prospective cohort study about sepsis epidemiology in critically ill adults from 17 ICUs of 16 regional central hospitals across China which encompassed a wide range of clinical settings and critical care populations.

## Methods

### Study setting and population

 The retrospective analysis based on database of a prospective cohort study about sepsis epidemiology sponsored by China Critical Care Sepsis Trial (CCCST) workgroup, which was performed in 17 Chinese ICUs between January 1st, 2014 and August 31st, 2015. A complete list of trial sites is provided in the Supplementary File. The protocol of the study was registered on August 3rd, 2013. The study was approved in all participating ICUs by their Hospital Human Ethics Committee. The chief ethics number was 2013FXHEC-KY2018. The registration number was ChiCTR-ECH-13,003,934. The study followed the ethical principles of the Declaration of Helsinki 1964. Informed consent from patients or their next of kin was obtained before patients joined in the study. All enrolled patients adhere to the following management principles: active treatment of primary disease and complications, and the same principles of treatment with antibiotics, nutritional metabolism and organ support.

We screened patients from the database of 4910 patients. Adult patients were eligible for inclusion if they stayed longer than 48 h in ICU, and none of the following exclusion criteria was fulfilled: (1) chronic kidney disease (CKD); (2) acquired insufficient data; (3) operated with nephrectomy or kidney transplantation; (4) developed AKI before ICU; (5) renal replacement therapy (RRT) for non-renal indications.

### Definitions and clinical endpoints

The definitions of AKI and AKI classification were depended on the serum creatinine and urine output criteria proposed by Kidney Disease: Improving Global Outcomes (KDIGO) [14]. New-onset AKI was defined as acute kidney injury occurred within 72hours after ICU admission. If a patient could be diagnosed with AKI at the time of admission (elevated serum creatinine or oliguria within the first 6 hours after admission that met the KDIGO criteria), it is considered that AKI was already occurred before ICU admission, and this patient couldn’t be diagnosed as new-onset AKI.CKD was defined according to the definition of National Kidney Foundation as estimated glomerular filtration rate (eGFR) < 60 ml/min/1.73 m^2^ for at least 3 months irrespective of the cause. GFR was estimated with the Cockcroft-Gault formula [15, 16]. The baseline creatinine was defined as follows: if at least five values were available the median of all values available from 6 months to 6 days prior to enrollment was used. Otherwise, the lowest value in the 5 days prior to enrollment was used. If no pre-enrollment creatinine was available or the emergency patient’ s serum creatinine was abnormal at the time of admission, the baseline creatinine was estimated using the Modification of Diet in Renal Disease (MDRD) equation assuming that baseline eGFR is 75 ml/min per 1.73 m^2^ [17]. The diagnosis of sepsis and septic shock was according to the sepsis 3.0 definition of the American College of Chest Physicians/ Society of Critical Care Medicine criteria [18]. Transient AKI was defined as kidney injury recovered within 48 h [19]. Persistent AKI was defined as acute kidney injury more than 48 h. Recovery of AKI was defined as the absence of any stage of AKI by either serum creatinine or urine output criteria. For example, a patient with stage 2 AKI would have to have a decrease in serum creatinine to less than 150 % of baseline, and be free of periods of oliguria longer than 6 h [20]. When 48 h follow-up was not possible due to death or discharge from ICU, AKI was defined as persistent if patients met KDIGO criteria for their last measurement, otherwise as transient. Weaning of RRT was defined as the cessation of RRT within 28 days, and requiring no further support for at least 7 days. Major surgery was defined as the surgery classification of grade 3 or 4 identified by the National Health Commission of China.

The primary endpoint was 28-day mortality. Secondary endpoints were weaning of RRT, length of ICU stay and hospital stay, hospital mortality.

### Data collection

Data were prospectively recorded during the hospital stay. The information collected included demographic characteristics, chronic illnesses, diagnosis, pre-ICU medications (whether or not used nephrotoxic drugs, nephrotoxic drugs included angiotensin converting enzyme inhibitors, non-steroidal anti-inflammatory drug, amikacin and amphotericin B) and treatment, the reason for ICU admission, whether or not infection, acute physiology and chronic health evaluation (APACHE II) (based on data recorded during the first 24 h of ICU admission), baseline serum creatinine, creatinine values every 12 h and hourly urine output on ICU admission and thereafter until transferred out of ICU, days of mechanical ventilation, use of vasoactive drugs, sequential organ failure assessment (SOFA) score and the value of lactate every day in the first 3 days after ICU admission. We also collected time of diagnosing AKI, initiation and weaning of RRT, ICU stay, hospital stay, hospital mortality and 28-day mortality.

### Statistical analysis

Continuous variables were presented as mean ± standard deviation (SD) or median values (25th and 75th percentiles), categorical variables were presented as percentiles. Continuous data between two groups was compared using the Student’s t-test or Mann-Whitney U tests, and categorical variables used the Chi-square test or Fisher’ s exact test. To assess the association of variables and new-onset AKI within the entire cohort, a logistic regression analysis was conducted to calculate the odds ratios (ORs) and 95 % confidence intervals (CIs). Regression models used a backward procedure with a significance level of 0.1. The 28-day survival rate was calculated using the Kaplan-Meier method. The survival comparison among the groups was performed using the log-rank test. Cox proportional hazards model was used to graphically describe the in-hospital survival. The hazard ratios (HRs) and 95 % confidence intervals for mortality rates were calculated using the Cox proportional hazard model after adjustment for potential confounders. For all analyses, statistical significance was indicated by two-sided *P* < 0.05. SPSS statistics 24 (IBM, Chicago, IL) and R 3.6.1 (R Project for Statistical Computing) were used for statistical analyses.

## Results

### Demographics

**Fig. 1:**
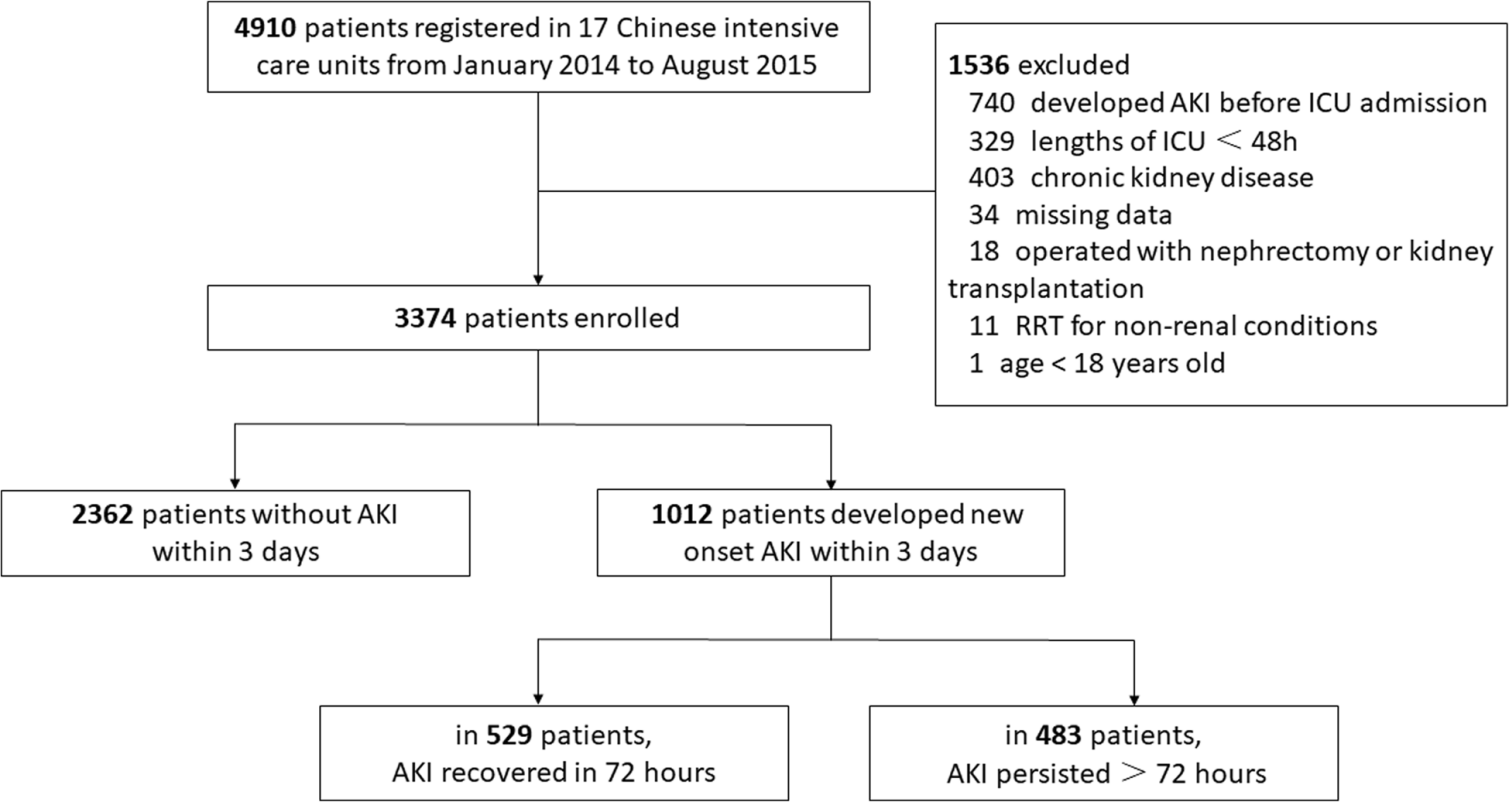
Study flow diagram. *AKI* acute kidney injury, *ICU* intensive care unit, *RRT* renal replacement therapy

We identified 3374 patients from 17 ICUs after applying all inclusion and exclusion criteria (Fig. 1). The median [interquartile range (IQR)] age of the patients was 64 (49–76) years. Of these, all were self-identified as Asian and 64 % were male. Patients mainly came from operation room (34.4 %, *n* = 1162), other department (31.7 %, *n* = 1071), emergency room (21.0 %, *n* = 709) and others (12.8 %, *n* = 432). The median APACHE ІІ score and SOFA score was 16 and 5 within 24 h after ICU admission, respectively. Sepsis was diagnosed at admission for 1183 (35.1 %) patients. 1912 (56.7 %) patients received mechanical ventilation. The median length of stay in hospital was 18 (11–27) days. The unadjusted in-hospital mortality rate was 20.5 % (*n* = 691). Table 1 listed all the baseline characteristics and covariates included in the regression models for the outcome of new-onset AKI and 28-day mortality.

**Table 1:** Baseline characteristics of patients stratified by new-onset acute kidney injury

Variables	All patients	No AKI	New onset AKI	*P* value
	*N*=3374	*N*=2362	*N*=1012	
Male *n* (%)	2144 (63.5)	1500 (63.5)	644 (63.6)	0.942
Age (years)	64 (49-76)	62 (48-75)	67 (52-79)	<0.001
BMI (kg/m^2^)	22.6 (20.0-24.5)	22.5 (19.8-24.5)	22.8(20.3-24.5)	0.136
Chronic comorbidities				
COPD/asthma *n* (%)	255 (7.6)	185 (7.8)	70 (6.9)	0.357
Coronary heart disease *n* (%)	542 (16.1)	342 (14.5)	200 (19.8)	<0.001
Hypertension *n* (%)	1054 (31.2)	663 (28.1)	391 (38.6)	<0.001
Diabetes *n* (%)	522 (15.5)	334 (14.1)	188 (18.6)	0.001
Cancer *n* (%)	369 (10.9)	251 (10.6)	118 (11.7)	0.378
Chronic liver disease *n* (%)	91 (2.7)	52 (2.2)	39 (3.9)	0.007
APACHEII	16 (10-22)	15 (9-20)	18 (13-25)	<0.001
SOFA	5 (3-8)	4 (3-7)	7 (4-10)	<0.001
Sepsis *n* (%)	1183 (35.1)	677 (28.7)	506 (50.0)	<0.001
Mechanical ventilation *n* (%)	1912 (56.7)	1330 (56.3)	582 (57.5)	0.519
Use of vasopressors *n* (%)	746 (22.1)	411 (17.4)	335 (33.1)	<0.001
Baseline creatinine (μmol/L)	83 (66-93)	82 (66-93)	84 (65-97)	0.268
Use of nephrotoxic drugs *n* (%)	362 (10.7)	195 (8.3)	167 (16.5)	<0.001
LOS in ICU (days)	6 (3-13)	6 (3-12)	7 (4-15)	<0.001
LOS in hospital (days)	18 (11-27)	18 (11-27)	18 (10-27)	0.377
Mortality				
ICU mortality	535 (15.9)	245 (10.4)	290 (28.7)	<0.001
28-day mortality	573 (17.0)	276 (11.7)	297 (29.3)	<0.001
Hospital mortality	691 (20.5)	346 (14.6)	345 (34.1)	<0.001

Continuous variables are presented as median and interquartile range

*BMI* body mass index, *COPD* chronic obstructive pulmonary disease, *APACHEII* acute physiologic and chronic health evaluationII, *SOFA* sequential organ failure assessment, *LOS* length of stay

### Incidence of new-onset AKI in different clinical settings

**Fig. 2:**
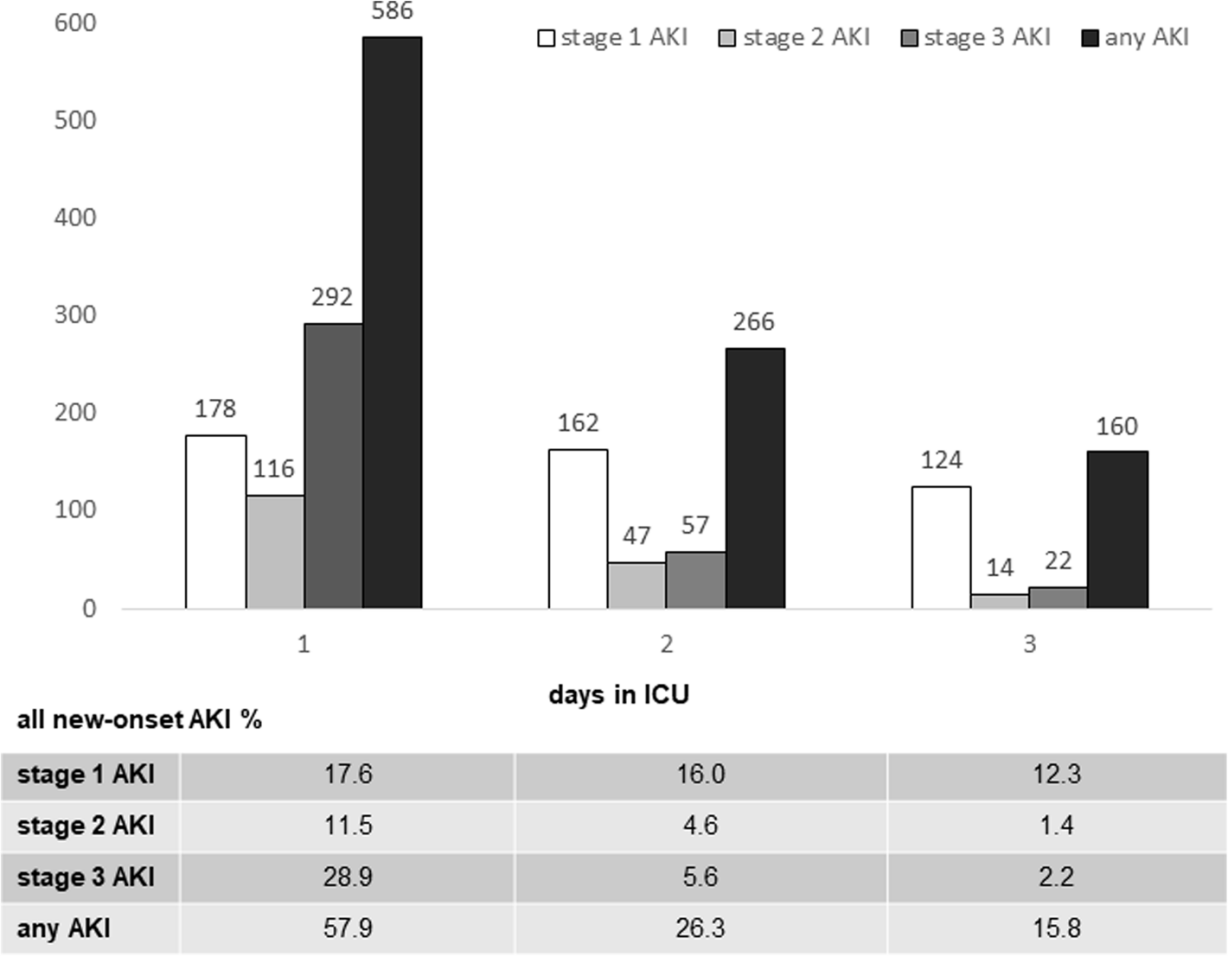
Histogram of new-onset AKI developed per day. *AKI* acute kidney injury, *ICU* intensive care unit

1174 (34.8 %) developed AKI in the first 7 days in ICU, among them, new-onset AKI (AKI occurred within 72 h after ICU admission) developed in 1012 (30.0 %) patients. 586 (57.9 %) of the new-onset AKI developed kidney injury on the first day of ICU. The number of new-onset AKI developed per day was shown in Fig. 2. The median SOFA score on the day of AKI diagnosis was 7 (4–10). Half of new-onset AKI patients had sepsis before the diagnosis of AKI, 286 (56.5 %) of them were septic shock. AKI severity according to the KDIGO classification was determined to be stage 1 in 464 (45.8 %) patients, stage 2 in 177 (17.5 %) patients, and stage 3 in 371 (36.7 %) patients. Among the new-onset AKI patients, 220 (21.7 %) patients received RRT. The most common causes of RRT were oliguria/anuria (*n* = 179, 81.4 %), followed by severe acidosis (pH < 7.15) (*n* = 79, 35.9 %), refractory hyperkalemia (K^+^ > 6 mEq/L) (*n* = 64, 29.1 %) and fluid overload with organ edema (*n* = 20, 9.1 %). 32.3 % (*n* = 71) of them were successfully weaning from RRT.

**Fig. 3:**
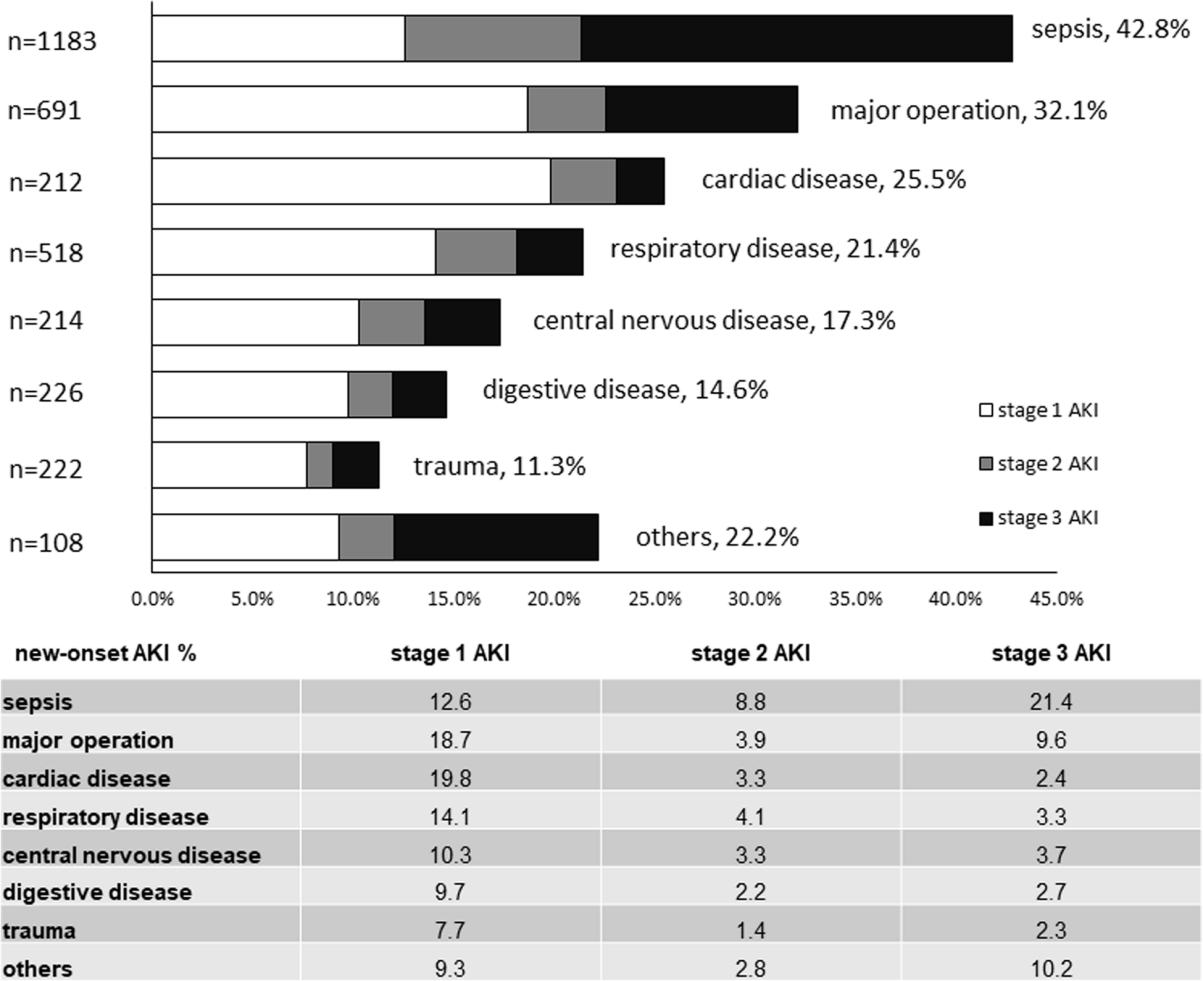
Number and incidence of new-onset AKI among patients transferred to ICU for different reasons. *AKI* acute kidney injury

The incidence of new-onset AKI varied among patients transferred to ICU for different reasons (Fig. 3). The three leading reasons with the highest incidence of new-onset AKI were sepsis (42.8 %), major surgery (32.1 %) and cardiac disease (25.5 %). Patients transferred to ICU due to sepsis had the highest incidence of new-onset AKI. Among them, stage 3 AKI accounted for 21.4 % of new-onset AKI, which is also the highest proportion among different populations.

### Risk factors for development of new-onset AKI

For all demographic data, clinical presentation data and laboratory findings presented in Table 1, we initially evaluated, using univariate analysis, each variable that displayed a statistically significant difference (*p* < 0.05) between new-onset AKI and non-AKI. On the univariate analysis, the risk factors associated with development of new-onset AKI were age, coronary heart disease, diabetes, hypertension, chronic liver disease, use of nephrotoxic drugs, sepsis, SOFA score, APACHEII score and use of vasopressors at ICU admission (Table 2). The above 10 variables were further processed using a multivariable logistic regression model, which selected eight variables that were predictive of new-onset AKI, including coronary heart disease, hypertension and chronic liver disease, use of nephrotoxic drugs, sepsis, SOFA score, APACHEII score and use of vasopressors.

**Table 2:** Risk factors for new-onset AKI

Variables	Crude OR (95 % CI)	Adjusted OR (95 % CI)	*P* value
Coronary heart disease	1.455 (1.200-1.764)	1.319 (1.053–1.652)	0.016
Chronic liver disease	1.781 (1.168–2.715)	1.641 (1.040–2.588)	0.033
Hypertension	1.613 (1.382–1.884)	1.312 (1.085–1.586)	0.005
Sepsis	2.489 (2.138–2.898)	1.636 (1.377–1.944)	0.000
SOFA	1.176 (1.151–1.202)	1.096 (1.063–1.130)	0.000
APACHE II	1.071 (1.060–1.081)	1.032 (1.020–1.045)	0.000
Use of nephrotoxic drugs	2.196 (1.760–2.741)	1.861 (1.461–2.370)	0.000
Use of vasopressors	2.349 (1.984–2.781)	1.268 (1.025–1.569)	0.029
Age	1.013 (1.009–1.018)	1.003 (0.998–1.008)	0.268
Diabetes	1.385 (1.139–1.686)	1.014 (0.812–1.266)	0.901

*AKI* acute kidney injury, *OR* odds ratio, *SOFA* sequential organ failure assessment, *APACHEII* acute physiologic and chronic health evaluationII

### New-onset AKI as an independent risk factor for death

**Fig. 4:**
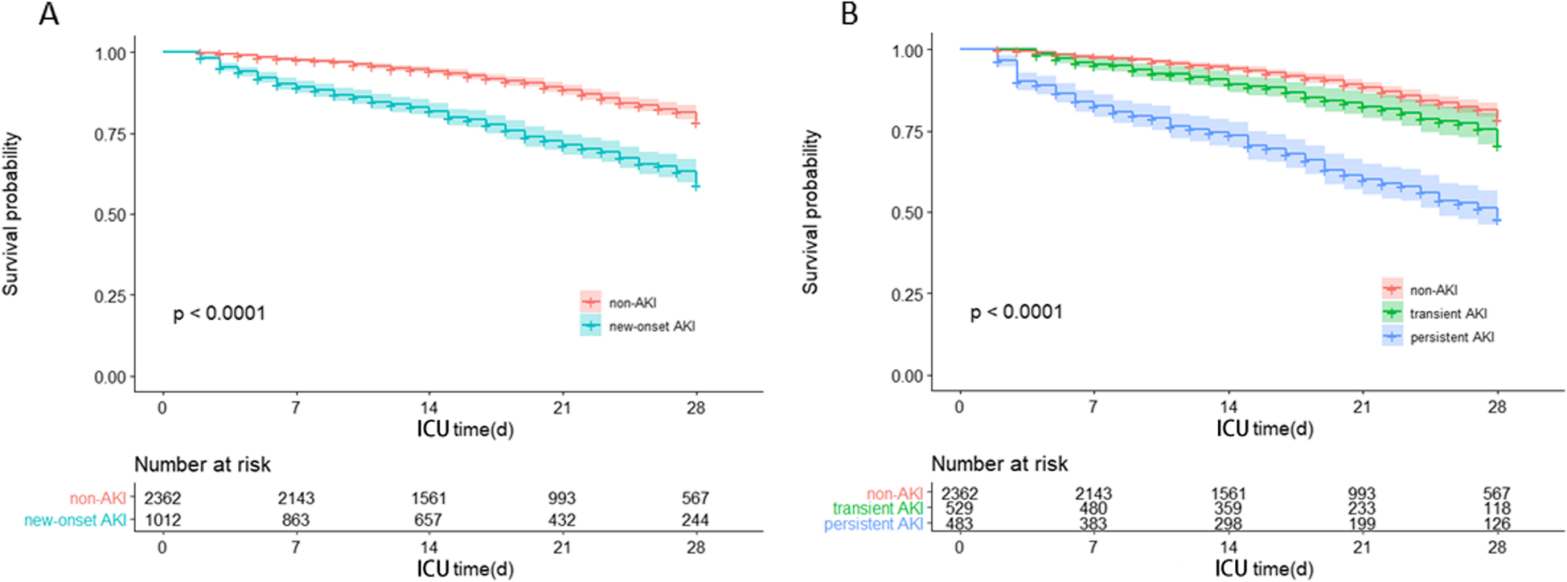
**a **Kaplan–Meier survival analysis comparing new-onset AKI and non-AKI groups.** b **Kaplan–Meier survival analysis comparing non-AKI, transient AKI and persistent AKI groups. *P* < 0.001 for all comparisons. Numbers of patients at risk at each time point shown below the graph. *AKI* acute kidney injury, *ICU* intensive care unit

**Fig. 5:**
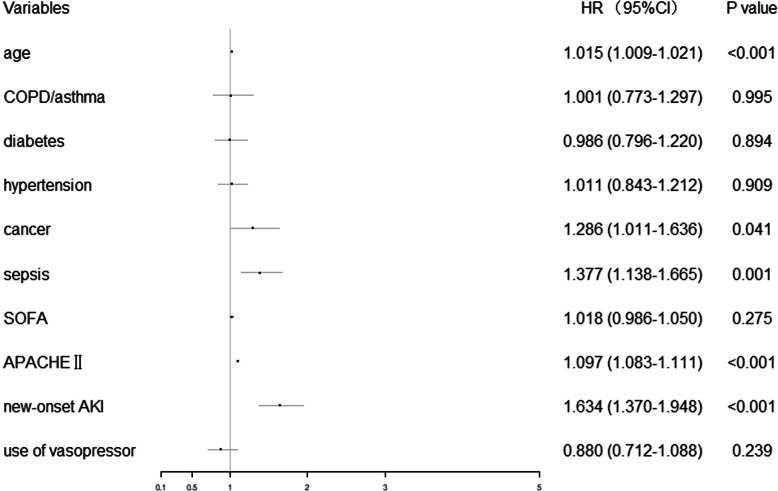
Forest plot of multivariable Cox proportional hazard regression analysis of new-onset AKI and 28-day mortality. *COPD* chronic obstructive pulmonary disease, *SOFA* sequential organ failure assessment, *APACHEII* acute physiologic and chronic health evaluationII, *AKI* acute kidney injury

573 (17.0 %) of enrolled patients had died within 28 days. Non-survivors were more likely to develop new-onset AKI than survivors. The 28-day mortality rate of the patients with new-onset AKI was higher than that of the non-AKI patients (*P* < 0.001). Patients were divided into two groups (new-onset AKI and non-AKI) for Kaplan-Meier survival curve analysis. The new-onset AKI group had a significantly lower 28-day survival rate than non-AKI groups (*P* < 0.001, Fig. 4 a). In our multivariate cox regression model (Fig. 5), after adjusting for age, comorbidities (COPD/asthma, hypertension, diabetes and cancer), APACHEII, SOFA score, the need for mechanical ventilation, the presence of sepsis and use of vasopressors, new-onset AKI (adjusted hazard ratio, 1.643; 95 % CI, 1.370–1.948; *P* < 0.001) was associated with a higher risk of 28-day mortality.

### Stratified analysis by duration of new-onset AKI

Of the new-onset AKI patients, 529 (52.7 %) patients with AKI recovered within 48 h, namely transient AKI, another 483 (47.3 %) patients were persistent AKI (AKI persisted more than 48 h). Baseline characteristics stratified by AKI duration were shown in Table S1. Patients who experienced persistent AKI were older and sicker, with higher APACHE II and SOFA scores, and were more likely to have sepsis and vasopressors needs. Table 2 presents outcomes stratified according to AKI duration. After adjustment for confounding covariates, we found that hypertension, baseline creatinine, APACHEII score, SOFA score, sepsis and use of vasopressors were associated with persistent AKI (Table 3). Compared to patients with transient AKI, patients with persistent AKI showed higher 28-day mortality, longer length of ICU and hospital stay (Table 4). Kaplan-Meier curve revealed significantly higher mortality rates in persistent AKI group compared to patients with transient AKI (*P* < 0.001; Fig. 4b). Survival probabilities at the 28th day were 88.3 % for patients without AKI, 82.0 % for those with transient AKI, and 58.2 % for those with persistent AKI. However, univariate cox proportional hazard model revealed that transient AKI was not associated with 28-day mortality (crude hazard ratio, 1.046, 95 % CI; 0.840–1.304, *P* = 0.686). For patients with new-onset AKI, univariate analysis revealed that persistent AKI was independently associated with higher mortality (crude hazard ratio, 2.483; 95 % CI, 1.945–3.169, *P* < 0.001). After adjustment for confounders, the association between persistent AKI and mortality was found to be qualitatively preserved (adjusted hazard ratio, 1.486; 95 % CI, 1.137–1.943; *P* < 0.001).

**Table 3:** Risk factors of persistent AKI

Variables	Crude OR (95 % CI)	Adjusted OR (95 % CI)	*P* value
Hypertension	1.406 (1.091–1.813)	1.412 (1.061–1.880)	0.018
Baseline creatinine (µmol/L)	1.005 (1.003–1.007)	1.002 (0.099–1.004)	0.163
APACHEII	1.094 (1.075–1.114)	1.057 (1.035–1.078)	0.000
SOFA at diagnostic day of AKI	1.210 (1.168–1.253)	1.117 (1.068–1.169)	0.000
Sepsis before diagnosis of AKI	3.784 (2.905–4.928)	2.579 (1.935–3.439)	0.000
Mechanical ventilaton	0.920 (0.685–1.236)		
Use of vasopressors	1.540 (1.184–2.005)	0.863 (0.626–1.191)	0.371

*AKI* acute kidney injury, *OR* odds ratio, *APACHEII* acute physiologic and chronic health evaluationII, *SOFA* sequential organ failure assessment

**Table 4:** Characteristics of new-onset AKI patients stratified by duration of acute kidney injury

	All new-onset AKI	Transient AKI	Persistent AKI
	*N*=1012	*N*=529	*N*=483
KDIGO stage			
Stage 1 *n* (%)	464 (45.8)	372 (70.3)	92 (19.0)
Stage 2 *n* (%)	177 (17.5)	76 (14.4)	101 (20.9)
Stage 3 *n* (%)	371 (36.7)	81 (15.3)	290 (60.0)
RRT	220	0	220
Causes of RRT			
Oliguria/anuria *n* (%)	179 (81.4)	0	179 (81.4)
Severe acidosis (pH < 7.15) *n* (%)	79 (35.9)	0	79 (35.9)
Refractory hyperkalemia (K^+^ > 6mmol/L) *n* (%)	64 (29.1)	0	64 (29.1)
Fluid overload with organ edema *n* (%)	20 (9.1)	0	20 (9.1)
SIRS with MODS *n* (%)	15 (6.8)	0	15 (6.8)
Others ^a^ *n* (%)	15 (6.8)	0	15 (6.8)
Weaning from RRT *n* (%)	71(32.3)	0	71(32.3)
LOS in ICU (days)	7 (4-15)	6 (3-11)	9 (5-17)
LOS in hospital (days)	18 (10-27)	18 (12-27)	17 (8-28)
28-day mortality *n* (%)	297 (29.3)	95 (18.0)	202 (41.8)

Continuous variables are presented as median and interquartile range

*AKI* acute kidney injury, *KDIGO* kidney disease improving global outcomes, *RRT* renal replacement therapy, *LOS* length of stay, *SIRS* systemic inflammatory response syndrome, *MODS* multiple organ dysfunction

^a^ signs and symptoms of uremia (bleeding, pericarditis and encephalopathy), azotemia (BUN > 100mg/dL), hypernatremia, high urea/creatinine levels

### Center variability

We observed intercenter variability in the rate of new-onset AKI [median, 27.4 %; IQR (18.0 − 36.5 %)], which showed the median rate was similar to the overall rate of new-onset AKI (30.0 %) and the 95 % confidence intervals overlap for the vast majority of sites, suggested the variability of each center did not cause a statistical difference. The rates for each individual center are shown in Table S2 in the Supplementary Appendix.

## Discussion

The detection rate of AKI based on serum creatinine change depends on the frequency of serum testing. However, some studies did not record the changes of creatinine before patients were transferred to ICU, which makes it impossible to accurately calculate the duration of kidney injury for some patients who already had AKI at admission. Patients with AKI before ICU often have serious illness condition, more complications and higher mortality. Therefore, in order to reduce the excessive confounding factors, we excluded the patients who developed AKI before admitted to ICU. Moreover, we excluded the patients diagnosed as AKI within the first 6 h after ICU admission, considering that the kidney injury at this time may actually occurred before ICU. We defined AKI that appeared in the first 72 h after ICU admission as new-onset AKI because the previous studies found that approximately 93.1 % of AKI occurred in the first 72 h after admitted to ICU in critically ill patients [5–7]. The definition of new-onset AKI in our study might prevent the influence of duration of AKI before ICU admission, which facilitated the precise grouping of transient AKI and persistent AKI. As we know, this study represented the largest and the most extensive analysis of new-onset AKI among ICU adults in China.

We calculated the incidence of new-onset AKI at 30.0 % among 3374 patients from 17 ICUs of 16 regional central hospitals encompassing a wide range of clinical settings. Our estimate was lower than the 57.3 % reported in EPI-AKI study [2], mainly because this study excluded patients with AKI before ICU admission and patients with CKD. We also assessed the incidence of new-onset AKI at 42.8 % among septic patients in our study. The prevalence of AKI in patients with severe sepsis or septic shock has been reported to be in the range of 30 − 40 % during the last two decades [21, 22], while the recent FINNAKI study showed 53 % of 918 patients with severe sepsis met the KDIGO criteria for AKI [23]. The main reason for the higher prevalence was that recent consensus definitions of AKI had increased the sensitivity of its detection. The prevalence of new-onset AKI among septic patients in our study was lower than FINNAKI study, this difference may represent the patients who have already had AKI before ICU admission. Logistic regression in our study showed that coronary heart disease, hypertension, chronic liver disease, use of nephrotoxic drugs, sepsis, SOFA score, APACHEII score and use of vasopressors were independent risk factors for new-onset AKI. These findings were consistent with previous findings [24, 25]. Sepsis was the most common contributing factor to new-onset AKI.

 The initiation of RRT among new-onset AKI patients reviewed the defects of traditional ways of treatment in China. Three leading reasons to start RRT were similar to the results of B.E.S.T. Kidney study [26], which showed the most common initiation of RRT was oliguria/ anuria, followed by high urea/creatinine, metabolic acidosis and fluid overload. However, only a few patients in our study started RRT because of high urea/creatinine, this may reflect Chinese doctors’ insensitivity to high urea/creatinine level. In addition, systemic inflammatory response syndrome (SIRS) with multiple organ dysfunction syndrome (MODS) was the initiation of RRT in some AKI patients, its proportion (6.8 %) is significantly higher than that of other countries although it might be the co-reason for RRT initiation [26]. This may be because some intensivists still believe that RRT is a remedy to reduce systemic inflammation.

Our study found that the new-onset AKI was associated with higher 28-day mortality and longer length of hospital stay. The patients with new-onset AKI had a probability for 28-day mortality that was 0.6 times higher compared with those without AKI, even after adjusting for a large number of possible confounders. However, only persistent AKI is associated with increased 28-day mortality rather than transient AKI. The prognostic impact of transient AKI remains controversy in previous studies [8, 10–12]. A large multicenter cohort study [11] demonstrated that both transient and persistent AKI were independently associated with a poor outcome, and that patients with persistent AKI had a higher risk of death than patients with transient AKI. Our results differ somewhat from previous studies [11, 12], which focused on all hospitalized patients. Our results are similar to a recent study of septic patients that also came to the conclusion that only persistent AKI is associated with significantly higher 28-day mortality [27]. The strong association between persistent AKI and the poor outcome can be explained as follows: the long duration of AKI indicates the exacerbated physiologic profiles and the poor prognosis. In addition, the pathophysiological mechanism of transient AKI and persistent AKI may be different. Transient AKI is considered to be “prerenal dysfunction”, while AKI that has not been recovered for a long time is more related to “acute tubular necrosis” [28]. Our results suggest for future clinical research to consider that quick and spontaneous resolving AKI may dilute trials and should not be the target of new treatment trials, and highlight the importance for clinician to put more value on duration of kidney injury. The uncertainty of illness makes it difficult for intensivists to predict transient and persistent AKI, but the several independent variables reported in Table 3 could help to distinguish persistent AKI. In our result, none of the transient AKI patient had RRT even though 15.3 % of them had stage 3 AKI (Table 4), and in clinical practice, RRT should be carefully considered to avoid unnecessary intervention for patients who are unlikely to develop persistent AKI.

The strengths of this study are the large sample size, the use of a consensus definition for AKI diagnosis, and actively searching for baseline serum creatinine, being available in the majority of the included patients, despite the high proportion of emergency admissions. On the other hand, this study also has limitations. First, patients with CKD were excluded. The prognosis of CKD is different from AKI with a normal baseline creatinine [29]. Furthermore, a patient with CKD has a high, abnormal and unstable creatinine level, the 0.3 mg/dl change cannot accurately reflect the development of AKI, and it may show an unimportant change of GFR. However, excluding CKD patients did make our results of AKI incidence lower than others. Second, as a retrospective analysis based on database of a prospective cohort study, we did not control the treatment of AKI, for AKI duration could be changed depending on the treatment. Third, our definition of persistent AKI was that kidney injury beyond 48 h from AKI onset, which was shorter than some previous studies [12, 30], this may reduce the severity of persistent AKI patients and influenced the 28-day mortality.

## Conclusions

In general ICU patients, 30.0 % of the patients developed new-onset AKI. The risk factors associated with new-onset AKI included coronary heart disease, hypertension, chronic liver disease, use of nephrotoxic drugs, sepsis, SOFA score, APACHEII score and use of vasopressors. The new-onset AKI was an independent risk factor for 28-day mortality. Transient AKI is not associated with 28-day death, while persistent AKI is strongly associated with worse outcome.

## Supplementary Information

**Additional file 1 **: **Table S1**. Baseline characteristics of new-onset AKI patients stratified by duration of acute kidney injury. **Table S2**. Characteristics and rates of new-onset AKI and 28-day death for each individual center.

## Data Availability

The datasets used and/or analyzed during the current study are available from the corresponding author on reasonable request.

## Abbreviations

AKI: Acute kidney injury
ICU: Intensive care unit
SOFA: Sequential organ failure assessment
APACHEII: Acute physiology and chronic health evaluationII
RRT: Received renal replacement therapy
HR: Hazard ratio
CI: Confidence interval
P: Probability
RIFLE: Risk, injury, failure, loss of kidney function, and end-stage renal disease
AKIN: Acute kidney injury network
KDIGO: Kidney disease improving global outcomes
CCCST: China Critical Care Sepsis Trial
eGFR: Estimated glomerular filtration rate
MDRD: Modification of Diet in Renal Disease
SD: Standard deviation
OR: Odds ratio
SIRS: Systemic inflammatory response syndrome
MODS: Multiple organ dysfunction syndrome
