# Supplementary material for: Risk factors, clinical features and outcome of new-onset acute kidney injury among critically ill patients: a database analysis based on prospective cohort study
Source: BMC Nephrol. 2021 Aug 25;22:289. doi: 10.1186/s12882-021-02503-x (PMC8390222; doi:10.1186/s12882-021-02503-x)
Supplement: Supplementary file 1 — Additional file 1 : Table S1. Baseline characteristics of new-onset AKI patients stratified by duration of acute kidney injury. Table S2. Characteristics and rates of new-onset AKI and 28-day death for each individual center. [file 12882_2021_2503_MOESM1_ESM.docx]

**Data Supplement**

**Risk factors, clinical features and outcome of new-onset acute kidney injury among critically ill patients: a database analysis based on prospective cohort study**

**Table S1.** Baseline characteristics of new-onset AKI patients stratified by duration of acute kidney injury

|  | Transient AKI | Persistent AKI | | |  |
| --- | --- | --- | --- | --- | --- |
|  | *N*=529 | *N*=483 | | | *P* value |
| Age (years) | 66 (52-78) | 69 (52-80) | | | 0.183 |
| Male *n* (%) | 339 (64.1) | 305 (63.1) | | | 0.757 |
| BMI (kg/m^2^) | 22.5 (20.3-24.2) | 22.9 (20.2-25.1) | | | 0.115 |
| Comorbidities |  |  |  |  |  |
| COPD/asthma *n* (%) | 39 (7.4) | 31 (6.4) | | | 0.550 |
| Coronary heart disease *n* (%) | 97 (18.3) | 103 (21.3) | | | 0.233 |
| Hypertension *n* (%) | 184 (34.8) | 207 (42.9) | | | 0.008 |
| Diabetes *n* (%) | 91 (17.2) | 97 (20.1) | | | 0.239 |
| Chronic liver disease *n* (%) | 20 (3.8) | 19 (3.9) | | | 0.899 |
| Cancer *n* (%) | 53 (10.0) | 65 (13.5) | | | 0.089 |
| Patient resource |  |  |  |  |  |
| Operation room *n* (%) | 204 (38.6) | 86 (17.8) | | | ＜0.001 |
| Other department *n* (%) | 167 (31.6) | 207 (42.9) | | | ＜0.001 |
| Emergency room *n* (%) | 104 (19.7) | 103 (21.3) | | | 0.512 |
| Other hospital *n* (%) | 50 (9.5) | 79 (16.4) | | | 0.001 |
| Other ICU *n* (%) | 4 (0.8) | 8 (1.7) | | | 0.186 |
| Use of nephrotoxic drugs *n* (%) | 94 (17.8) | 73 (15.1) | | | 0.256 |
| Baseline creatinine (umol/L) | 80 (64-95) | 85 (67-103) | | | 0.006 |
| APACHEⅡ | 16 (11-21) | 22 (16-28) | | | ＜0.001 |
| SOFA at diagnostic day of AKI | 5 (3-8) | 9 (6-11) | | | ＜0.001 |
| Sepsis before AKI diagnosis *n* (%) | 217 (41.0) | 350 (72.5) | | | ＜0.001 |
| Mechanical ventilation *n* (%) | 328 (62.0) | 254 (52.6) | | | 0.002 |
| Use of vasopressors *n* (%) | 151 (28.5) | 184 (38.1) | | | 0.001 |
| LOS in ICU (days) | 6 (3-11) | 9 (5-17) | | | ＜0.001 |
| LOS in hospital (days) | 18 (12-27) | 17 (8-28) | | | 0.083 |
| ICU mortality *n* (%) | 86 (16.3) | 204 (42.2) | | | ＜0.001 |
| 28-day mortality *n* (%) | 95 (18.0) | 202 (41.8) | | | ＜0.001 |
| Hospital mortality *n* (%) | 108 (20.4) | 237 (49.1) | | | ＜0.001 |

Continuous variables are presented as median and interquartile range

*AKI* acute kidney injury, *BMI* body mass index, *COPD* chronic obstructive pulmonary disease, *ICU* intensive care unit, *APACHEⅡ* acute physiologic and chronic health evaluationⅡ, *SOFA* sequential organ failure assessment, *LOS* length of stay.

**Table S2.** Characteristics and rates of new-onset AKI and 28-day death for each individual center

| Center | Hospital | Province/city | All patients  *N* | Enrolled patients  *N* | age | APACHEⅡ | SOFA | New-onset AKI  *N* (%) | Death at 28-day  *N* (%) |
| --- | --- | --- | --- | --- | --- | --- | --- | --- | --- |
| Department of Critical Care Medicine | Fuxing Hospital | Beijing | 285 | 225 | 80 (68-86) | 19 (15-25) | 5 (3-9) | 102 (45.3) | 87 (38.7) |
| Department of Critical Care Medicine | West China Hospital | Sichuan | 867 | 771 | 60 (47-73) | 20 (16-25) | 7 (5-10) | 211 (27.4) | 168 (21.8) |
| Medical Intensive Care Unit | Peking Union Medical College Hospital | Beijing | 765 | 510 | 59 (41-70) | 18 (14-24) | 5 (3-8) | 186 (36.5) | 111 (21.8) |
| Department of Critical Care Medicine | Guangdong General Hospital | Guangdong | 343 | 289 | 63 (51-74) | 10 (7-16) | 4 (2-7) | 108 (37.4) | 30 (10.4) |
| Department of Critical Care Medicine | The First Affiliated Hospital of China Medical University | Shenyang | 333 | 301 | 71 (61-79) | 10 (8-13) | 4 (2-6) | 83 (27.6) | 21 (7.0) |
| Surgical Intensive Care Unit | Zhongshan Hospital | Shanghai | 251 | 239 | 65 (56-74) | 9 (6-13) | 2 (1-4) | 39 (16.3) | 10 (4.2) |
| Intensive Care Unit | The First Hospital of Jilin University | Changchun | 219 | 184 | 58 (37-71) | 14 (10-22) | 5 (3-7) | 43 (23.4) | 27 (14.7) |
| Department of Critical Care Medicine | China-Japan Friendship Hospital | Beijing | 270 | 219 | 74 (58-82) | 15 (11-19) | 4 (3-6) | 72 (32.9) | 41 (18.7) |
| Department of Critical Care Medicine | Beijing Friendship Hospital | Beijing | 199 | 164 | 67 (51-79) | 15 (10-21) | 5 (3-9) | 61 (37.2) | 28 (17.1) |
| Surgical Intensive Care Unit | Beijing Chaoyang Hospital | Beijing | 15 | 10 | 71 (61-83) | 12 (10-15) | 3 (2-4) | 2 (20) | 0 (0.0) |
| Department of Respiratory and Critical Care Medicine | Beijing Chaoyang Hospital | Beijing | 141 | 108 | 70 (53-81) | 12 (8-15) | 4 (2-5) | 35 (32.4) | 15 (13.9) |
| Department of Critical Care Medicine | General Hospital of Ningxia Medical University | Ningxia | 179 | 150 | 58 (46-71) | 16 (11-19) | 5 (3-8) | 27 (18.0) | 16 (10.7) |
| Department of Critical Care Medicine | Xiangya Hospital | Changsha | 96 | 78 | 56 (44-67) | 10 (5-14) | 3 (2-5) | 16 (20.5) | 6 (7.7) |
| Department of Critical Care Medicine | Beijing Tongren Hospital | Beijing | 102 | 87 | 65 (50-79) | 12 (8-15) | 3 (2-6) | 14 (16.1) | 7 (8.0) |
| Department of Critical Care Medicine | Peking University Third Hospital | Beijing | 22 | 19 | 73 (53-80) | 19 (16-27) | 8 (6-11) | 11 (57.9) | 2 (10.5) |
| Surgical Intensive Care Unit | Xuanwu Hospital | Beijing | 8 | 7 | 67 (42-70) | 4 (3-11) | 3 (2-4) | 0 (0.0) | 1 (14.3) |
| Department of Critical Care Medicine | Beijing Tiantan Hospital | Beijing | 20 | 13 | 60 (55-71) | 14 (10-21) | 3 (2-5) | 2 (5.4) | 3 (23.1) |
